# Clarification and transparency on ILR migration review

**DOI:** 10.1016/j.ahjo.2025.100537

**Published:** 2025-04-01

**Authors:** Allam Harfoush

**Affiliations:** University of Chester, United Kingdom

Thank you for sharing your concerns [1]. I appreciate the opportunity to address these points and provide further clarification.

My review [2] began after reading a specific case report on ILR migration, which sparked my interest in exploring its occurrence and contributing factors. This led me to conduct an extensive literature search to identify relevant cases.

To ensure a comprehensive review, I used multiple databases, including PubMed, Cochrane, and CINAHL, as well as reference lists of related articles. I also reviewed preprints to capture the most current data, though I did not find any recent preprints directly related to this topic. It was only recently (30-03-2025) that I became aware of your group's work on this subject.

I have great respect for your research and the valuable contributions your team has made in this field. My methodology was independently developed, and I took great care to ensure originality. This review employed a systematic approach to meta-summarise case reports, focusing on a thorough review of these cases.

As researchers, we share a common goal of advancing scientific knowledge, and I fully uphold the principles of ethical research. If further discussion is needed to clarify any remaining concerns, I am more than happy to engage in dialogue. Additionally, I will inform the editorial boards of *American Heart Journal Plus: Cardiology Research and Practice* to ensure all relevant parties are aware of the situation and proper acknowledgment is made.

Thank you again for bringing this to my attention. Please do not hesitate to reach out if there is any additional information I can provide.

Kind regards

Dr A. Harfoush

30/03/2025

## CRediT authorship contribution statement

**Allam Harfoush:** Writing – original draft.

## Declaration of competing interest

The authors declare that they have no known competing financial interests or personal relationships that could have appeared to influence the work reported in this paper.
